# Glial cell line derived neurotrophic factor (GDNF) induces mucosal healing via intestinal stem cell niche activation

**DOI:** 10.1111/cpr.13758

**Published:** 2024-11-28

**Authors:** Marius Hörner, Natalie Burkard, Matthias Kelm, Antonia Leist, Thekla Selig, Catherine Kollmann, Michael Meir, Christoph Otto, Christoph‐Thomas Germer, Kai Kretzschmar, Sven Flemming, Nicolas Schlegel

**Affiliations:** ^1^ Department of General, Visceral Vascular and Pediatric Surgery University Hospital Wuerzburg Wuerzburg Germany; ^2^ Mildred Scheel Early Career Centre for Cancer Research Wuerzburg University Hospital Wuerzburg, MSNZ/IZKF Wuerzburg Germany

## Abstract

Mucosal healing is critical to maintain and restore intestinal homeostasis in inflammation. Previous data provide evidence that glial cell line‐derived neurotrophic factor (GDNF) restores epithelial integrity by largely undefined mechanisms. Here, we assessed the role of GDNF for mucosal healing. In dextran sodium sulphate (DSS)‐induced colitis in mice application of GDNF enhanced recovery as revealed by reduced disease activity index and histological inflammation scores. In biopsy‐based wounding experiments GDNF application in mice improved healing of the intestinal mucosa. GDNF‐induced epithelial recovery was also evident in wound assays from intestinal organoids and Caco2 cells. These observations were accompanied by an increased number of Ki67‐positive cells in vivo after GDNF treatment, which were present along elongated proliferative areas within the crypts. In addition, the intestinal stem cell marker and R‐spondin receptor LGR5 was significantly upregulated following GDNF treatment in all experimental models. The effects of GDNF on cell proliferation, LGR5 and Ki67 upregulation were blocked using the RET‐specific inhibitor BLU‐667. Downstream of RET‐phosphorylation, activation of Src kinase was involved to mediate GDNF effects. GDNF promotes intestinal wound healing by promoting cell proliferation. This is mediated by RET‐dependent activation of Src kinase with consecutive LGR5 upregulation, indicating activation of the stem cell niche.

## Graphical abstract

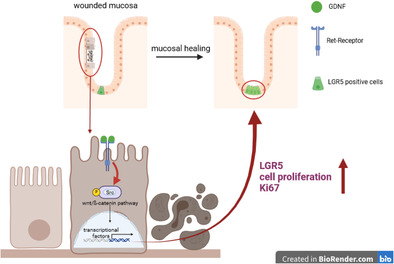

## 1 INTRODUCTION

Mucosal healing is a critical prerequisite to promote clinical recovery of patients suffering from intestinal inflammation, including inflammatory bowel diseases (IBD). This is reflected by current therapy guidelines for IBD patients that recommend the induction of mucosal healing as an important therapeutic aim, as this usually precedes clinical remission.^1^, ^2^

Intestinal epithelial wound healing is a complex process in which cell proliferation, migration, intercellular adhesion and cellular differentiation are required.^3^ In addition, the intestinal stem cell niche as the source for epithelial cell self‐renewal is crucial for ensuring homeostasis, repair and regeneration of the intestinal epithelium.^4^ Intestinal epithelial stem cells are marked by Leucin‐rich repeat‐containing G‐protein coupled receptor 5 (LGR5). LGR5 is stimulated by R‐spondins leading to the recruitment of LRP‐frizzled receptor complex to induce Wnt‐signalling, which is a key driver for intestinal cell proliferation.^5^, ^6^ For successful mucosal healing, the resolution of the inflammatory response is thought to be one important step. In addition, factors that promote the regeneration of intestinal epithelial cells are required and need to be identified to develop novel therapies targeting mucosal healing.

Previous data suggest that Glial cell line‐derived neurotrophic factor (GDNF) could be one of the factors to improve mucosal healing in the gut.^7^, ^8^, ^9^ Smooth muscle cells, enteric glial cells and epithelial cells are considered to provide relevant amounts of GDNF in the intestine.^10^, ^11^, ^12^ Previous data provided evidence that GDNF restores the loss of intestinal epithelial barrier function under inflammatory conditions via RET receptor activation.^9^ In addition, GDNF induces intestinal cell differentiation by stabilising intercellular junctional proteins in murine organoids and intestinal epithelial cell lines.^12^ Based on cell culture experiments it has been suggested that GDNF induces epithelial wound healing by yet undefined mechanisms. However, the significance of this observation in vivo and the potential involvement of RET‐dependent signalling remains largely unclear. It is important to note that in drosophila, RET‐induced signalling has been observed to promote Wnt activation in intestinal progenitor cells to sustain proliferation.^13^ In line with this, a previous study suggested that the regeneration of enterochromaffin cells are promoted by GDNF/RET‐induced proliferation.^14^ All these observations led to the hypothesis that GDNF‐mediated RET activation could promote intestinal mucosal regeneration by the induction of cell proliferation. To test this, we used a mouse model of DSS‐induced colitis and biopsy‐based mucosal wound assays to evaluate whether GDNF application affects mucosal healing and regeneration in vivo. Furthermore, differentiated Caco2 cells and murine and human intestinal organoids were used to identify the associated mechanisms and the potential role of RET‐dependent signalling.

## 2 RESULTS

### 2.1 GDNF application resulted in augmented recovery in DSS colitis

**FIGURE 1:**
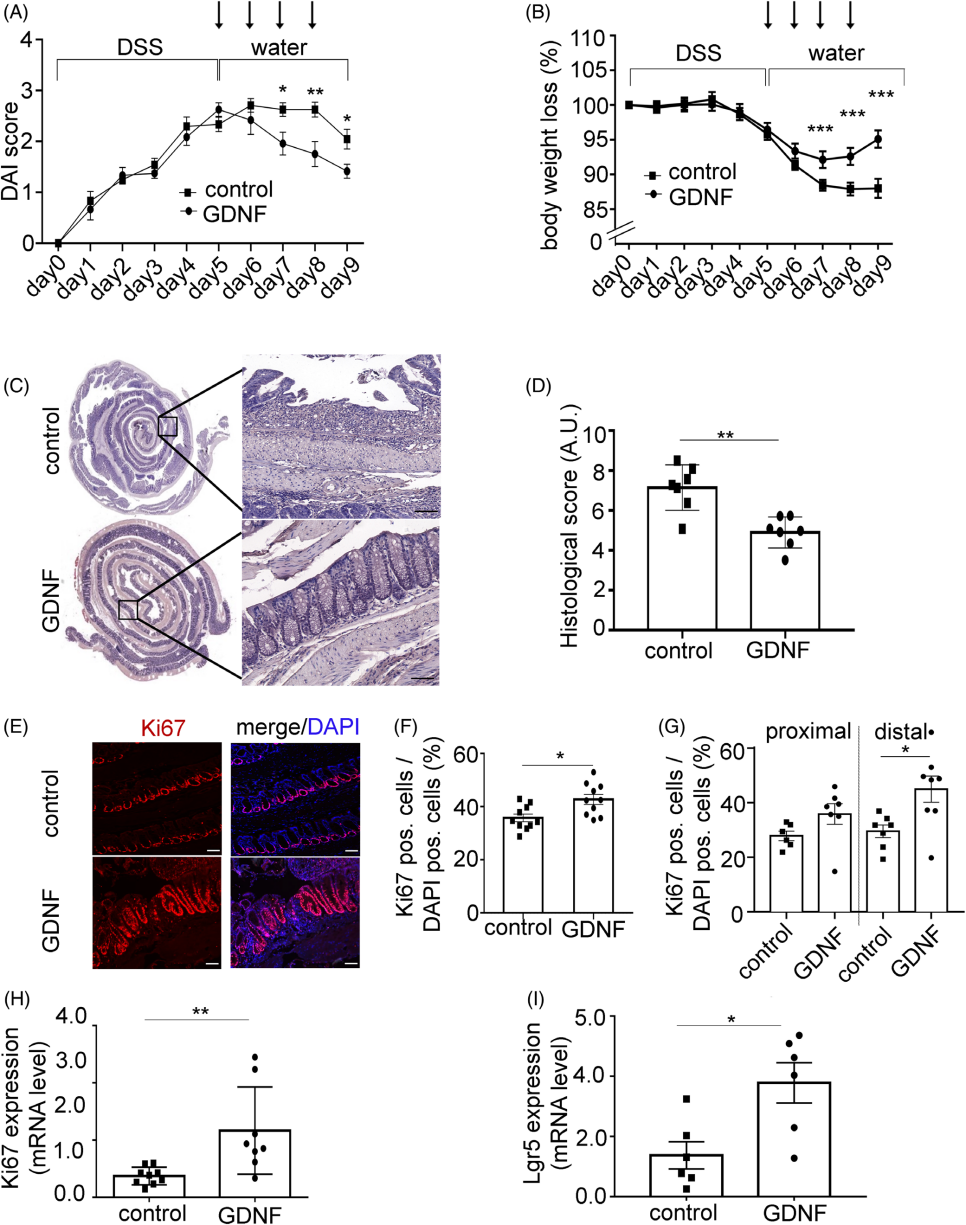
GDNF enhances mucosal recovery after DSS‐treatment. (A, B) Disease activity index (DAI) and changes of body weight loss over nine days during the DSS colitis experiment (control group *n* = 13; GDNF‐treated group *n* = 13). The arrows indicate the days on which GDNF or NaCl was administered intraperitoneally. Data are are expressed as means ± SEM. Significance was determined by 2‐way ANOVA followed by Bonferroni's posttest. *p ≤ 0.05, **p ≤ 0.01; ***p ≤ 0.001. (C) Representative H&E images of sections of Swiss roll mounts from the distal colon of DSS‐treated mice injected intraperitoneally with either GDNF or NaCl. Colonic tissues were harvested at day 10 of the experiment. Inset display magnifications from the areas marked in the overview. Scale bar 100 μm.(D) Histological injury score shows reduced mucosal ulcerations in GDNF‐treated mice compared with controls. Dots represent individual mice (*N* = 7 DSS + GDNF; *n* = 7 DSS + NaCl). Data represent are expressed as mean ± SEM. Significance is determined by a 2‐tailed Student's *t*‐test **p ≤ 0.01. (E) Representative images from Swiss role mounts with anti‐Ki67 (red) and/or DAPI (blue) for control condition (NaCl i.p.) and following GDNF treatment. Scale bar 50 μm. (F) Quantification of proliferative cells (Ki67‐positive)/DAPI‐positive is shown as a ratio determined from Swiss roll staining is presented. Dots represent the mean value calculated from the analysis of the swiss rolls from individual mice (*n* = 8 DSS + NaCl; *n* = 8 DSS + GDNF). Data are means ± SEM Significance is determined by a 2‐tailed Student's *t*‐test. *p ≤ 0.05. (G) Figure G shows the number of Ki67‐positive/DAPI‐positive cells in proximal and distal colon. Dots represent the mean value calculated from the analysis of the swiss rolls from individual mice (*n* = 7 DSS + NaCl; *n* = 7 DSS + GDNF). Data are means ± SEM Significance is determined by a 2‐tailed Student's *t*‐test. *p ≤ 0.05. (H) qRT‐PCR data from colon tissue lysates are shown for Ki67 expression or expression of intestinal stem cell marker *Lgr5* (I) are shown. Data are means ± SEM. Significance is determined by a 2‐tailed Student's *t*‐test. *p ≤ 0.05. GDNF, glial cell line‐derived neurotrophic factor.

The DSS colitis recovery model was used to assess the potential effects of GDNF on intestinal epithelial integrity and overall recovery of animals with intestinal inflammation. Application of 2.5% DSS resulted in increased DAI and weight loss after 5 days (Figure 1A,B). Daily i.p. application of GDNF (5 μg/kg bodyweight in 100 μL NaCl 0.9%) after colitis‐induction significantly decreased disease severity and increased body weight compared to control animals. H&E staining of the colon showed more severe inflammation in control animals, as revealed by increased immune cell infiltration and mucosal injury, compared to animals treated with GDNF, where inflammation was less pronounced with lower rates of inflammatory cells in the gut wall and increased restoration of epithelial integrity (Figure 1C). This was reflected by a significantly lower histological injury score (Figure 1D) of 4.89 ± 0.76 in contrast to control animals with 7.15 ± 1.14 (Figure 1D).

Colon sections were stained for DAPI and Ki67 to further analyse the influence of GDNF on intestinal cell proliferation (Figure 1E). In control animals, Ki67‐positive cells were visualised at the bottom of the crypts. This was significantly changed in GDNF‐treated animals with colon crypts appeared elongated and with significantly increased number of Ki67‐positive cells of 42.73 ± 4.88% compared to 35.76 ± 3.56% in control (*p* < 0.05; Figure 1F). To identify potential differences between the distal and proximal colon, we analysed Ki67‐positive cells in the distal and proximal colon seperately. This showed an increased number of Ki67‐positive cells in the distal colon sections after GDNF application of 44.95 ± 5.71% compared to the control animals of 29.5 ± 3.61% (*p* < 0.05, Figure 1G). In proximal colon sections, GDNF application increased the number of Ki67‐positive cells to 35.85 ± 4.61% compared to 27.82 ± 3.13% in the control (*p* = 0.45, Figure 1G). This showed a stronger effect GDNF on cell proliferation in the distal than in the proximal part of the colon. In qRT‐Polymerase chain reaction (PCR) measurements from colon tissue samples, *Mki67* mRNA levels were significantly augmented in GDNF‐treated animals to 2.5 ± 0.54‐fold of controls (*p* < 0.01; Figure 1H). This was paralleled by a significantly increased expression of *Lgr5* in qRT‐PCR measurements following GDNF treatment (3.5 ± 0.67‐fold of control; *p* < 0.05; Figure 1I).

### 2.2 GDNF increases mucosal healing in vivo as revealed by biopsy‐based wounding assays

**FIGURE 2:**
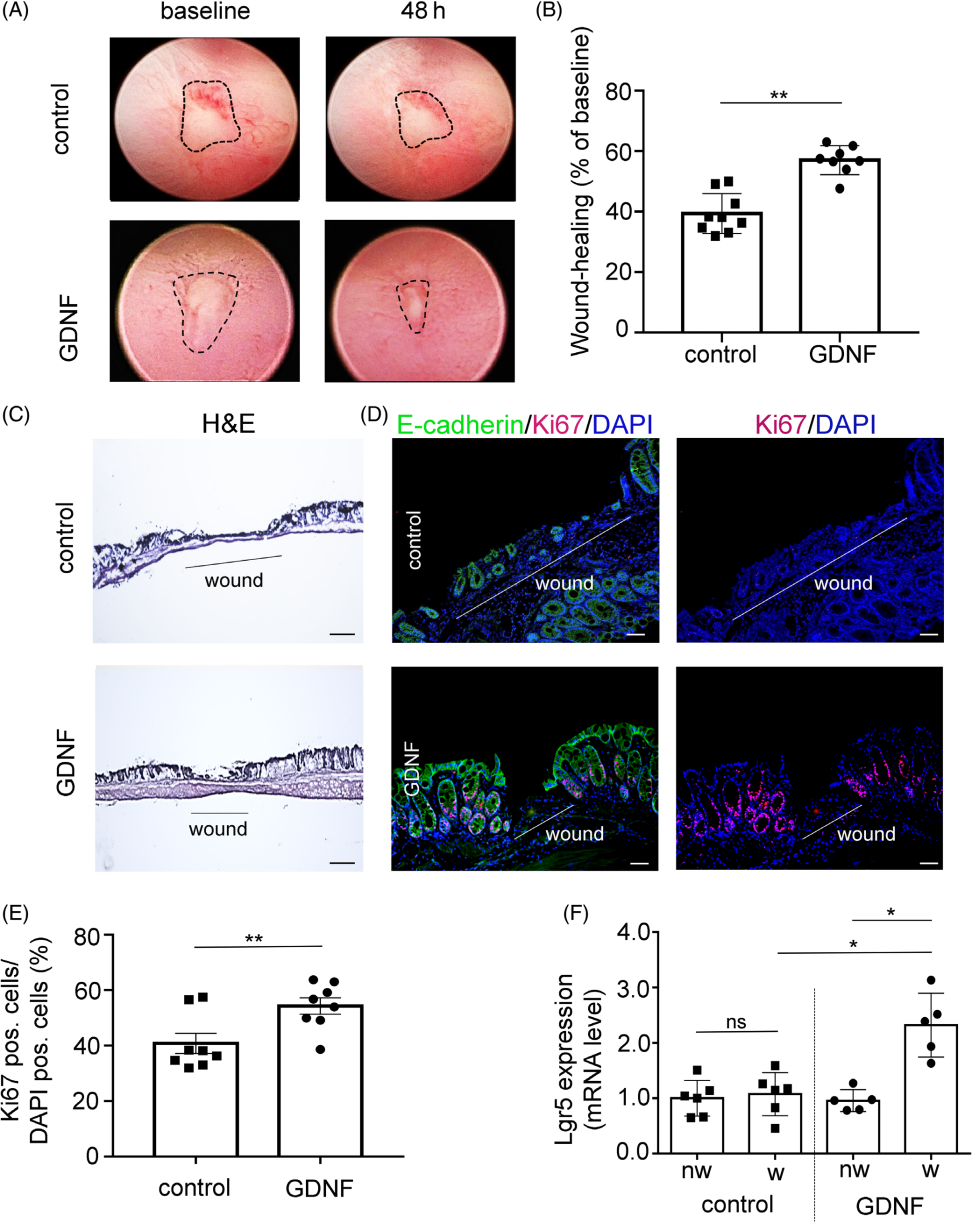
Enhanced wound healing in colonoscopy experiments mediated through increased cell proliferation and Lgr5 expression. (A) Representative endoscopic images of biopsy‐induced colonic wounds at 24 h (baseline) and 72 h postinjury that is, 48 h after baseline assessment in GDNF‐treated and control mice are shown. (B) Quantification of the wound surface at 24 h (baseline) and after 48 h revealed significantly increased wound healing in GDNF‐treated mice compared to control. Dots represent the mean value within three to five wounds from individual mice. Data are combined values of two independent experiments and are expressed as mean ± SEM. Significance is determined by two‐tailed Student's *t*‐test. **p ≤ 0.01. (C) Representative images of H&E staining of wound sections from the distal colon of mice treated with either GDNF or NaCl are shown. Scale bar 100 μm. (D) Representative immunofluorescence images with proliferation marker Ki67 (red), E‐cadherin (green) in wound‐adjacent epithelial crypts after 48 h of treatment with GDNF. Nuclei are stained in blue (DAPI). Scale bar is 50 μm. (E) Quantification of Ki67‐positive cells in wound‐adjacent crypts after treatment with GDNF or NaCl is shown. Data are shown as mean ± SEM (*n* = 8 experiments) and were analysed by 1‐way ANOVA followed by Tukey's post hoc testing *p* ≤ 0.01. (F) Expression of the Lgr5 stem cell marker in biopsy‐induced wounds is shown in qRT‐PCR. The left columns show animals treated with NaCl, whilst the right columns show animals treated with GDNF. The term ‘nw’ (non‐wounded) refers to samples outside the wound area, whilst ‘w’ (wounded) indicates expression within the wound site. Data are shown as mean ± SEM (*n* = 5–6 experiments) and were analysed by 1‐way ANOVA followed by Tukey's post hoc testing. *p ≤ 0.05. GDNF, glial cell line‐derived neurotrophic factor.

Next, we assessed the effect of GDNF on mechanically induced wounds in vivo using a well‐characterised colonoscopy‐based wound healing assay. The mucosal wound area was assessed up to 72 h after wounding, and final wound closure was compared with the area at 24 h (referred to as baseline) (Figure 2A). Under control conditions, wound closure was 39.35 ± 5.24% of the wounded area in the time course of experiments (Figure 2B). GDNF application significantly accelerated wound healing compared to controls with a wound closure of 57.05 ± 4.89% of the wounded area (*p* < 0.05). H&E staining of sections of the wounded areas demonstrated more pronounced epithelial recovery in GDNF‐treated wounds compared to controls (Figure 2C). This was confirmed by immunostaining with E‐cadherin of cells in crypts and the mucosal surface (Figure 2D). Comparable to the observations in DSS‐induced colitis, co‐staining with Ki67 demonstrated a significantly increased number of proliferative cells in the crypts following GDNF treatment compared to controls. This was verified by quantification of the relative amount of Ki67‐positive cells in relation to all cell nuclei visualised by DAPI when GDNF treatment resulted in an increased rate of Ki67‐positive cells of 55.29 ± 6.71% compared to 38.15 ± 5.64% under control conditions (*p* < 0.01; Figure 2E). In qRT‐PCR, the mRNA expression of intestinal stem cell marker *Lgr5* was comparable in punch biopsies from wounded areas compared to samples from healthy tissue in control animals. In contrast, mRNA expression of *Lgr5* in punch biopsies from wounded areas in GDNF‐treated animals was 2.27 ± 0.54‐fold (*p* < 0.05) increased compared to punch biopsies of non‐wounded mucosa from the same animals (Figure 2F). These experiments demonstrated that GDNF‐induced upregulation of *Lgr5* results in significantly increased cell proliferation and intestinal wound healing in conditions of mucosal injury and repair in vivo.

### 2.3 GDNF‐induced upregulation of LGR5 and increased wound healing in differentiated human organoids

**FIGURE 3:**
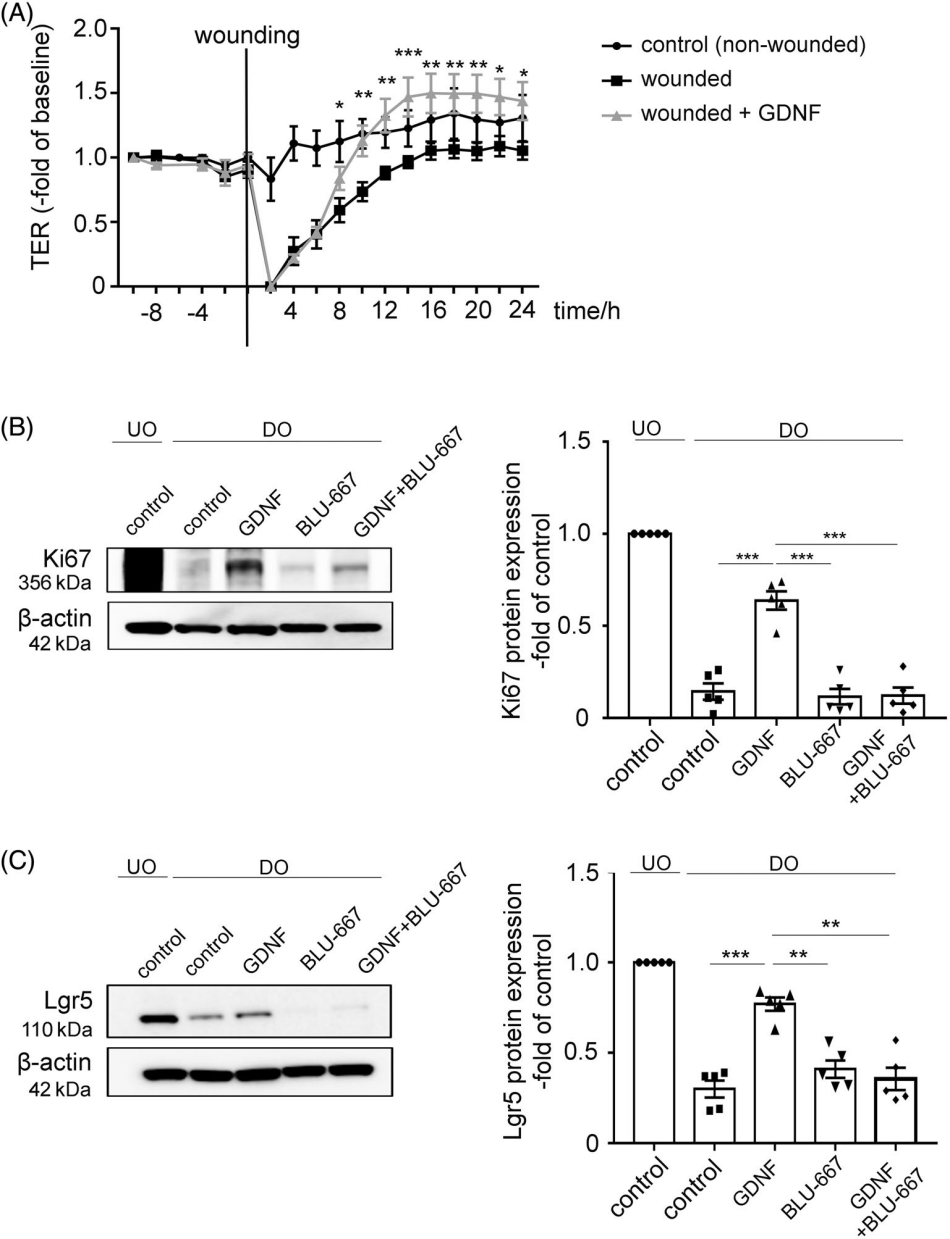
GDNF effect in human organoids mediated through Ret‐receptor signalling, enhanced LGR5 expression and cell proliferation. (A) TER wounding of 2D intestinal human organoids on electrode arrays resulted complete loss of TER after wounding. GDNF application increased recovery of TER compared to controls. Unwounded controls showed stable TER values in the timecourse of measurements (*n* = 4 experiments from four different patient‐derived organoids at different passages). Significant differences between the control group and the GDNF‐treated group is observed beginning after 8 h. Data shown as mean ± SEM and were analysed by 1‐way ANOVA followed by Tukey's post hoc testing. *p ≤ 0.05. (B) Representative Western blots from human organoids for Ki67 is shown (left). Membranes were reprobed with β‐actin as loading control (*n* = 5). UO = undifferentiated organoids; DO = differentiated organoids. Western blot quantifications present data as relative protein levels compared to β‐actin (OD of protein of interest/OD of β‐ actin) normalised to untreated controls of undifferentiated organoids. Quantification confirms increased levels of Ki67 under GDNF treatment. Data are shown as mean ± SEM (*n* = 5 experiments) and were analysed by 1‐way ANOVA followed by Tukey's post hoc testing. ***p ≤ 0.001. (C) Representative Western blots from human organoids Lgr5 and β‐actin as loading control under the different conditions are shown (*n* = 5; left). UO = undifferentiated organoids; DO = differentiated organoids. Western blot quantifications present data as relative protein levels compared to β‐actin (OD of protein of interest/OD of β‐ actin) normalised to untreated controls of undifferentiated organoids. Quantification confirms increased levels of Lgr5 under GDNF treatment. Data are shown as mean ± SEM (*n* = 5 experiments) and were analysed by 1‐way ANOVA followed by Tukey's post hoc testing. **p ≤ 0.01, ***p ≤ 0.001. GDNF, glial cell line‐derived neurotrophic factor; TER, transepithelial electrical resistance.

To validate the results of in vivo experiments in mice, human intestinal organoids were used to assess the relevance of the GDNF effects observed in mice in human epithelium.Two‐dimensionalized human organoid monolayers were cultivated on electrodes followed by electroporation of the cell monolayers which results in a defined wounding area and immediate loss of TER. Following application of a current of 2000 μA, for 10 s, 40000 Hz to induce electrical wounding, TER dropped completely to 0% of baseline. In the following TER recovered to baseline levels in the time course of 24 h in untreated organoid monolayers suggesting epithelial wound healing. Application of 100 ng/mL GDNF improved wound healing as early as 8 h after wounding compared to untreated wounded controls (0.46 ± 0.15‐fold compared to 0.95 ± 0.14‐fold, *p* < 0.05) (Figure 3A).

Based on the observation of LGR5 upregulation in mucosal tissue lysates in vivo we tested next whether GDNF is effective to activate the stem cell niche in human organoids. To test this, human organoids were differentiated by Wnt‐depletion as described previously.^15^ This led to a reduced Ki67 and LGR5 expression after 5 days of Wnt‐depletion as revealed by Western blot analyses (Figure 3B). Comparable to the in vivo experiments, stimulation of differentiated human organoids with GDNF increased both, Ki67 (from 0.14 ± 0.06‐fold of UD controls to 0.64 ± 0.05‐fold of UD controls, *p* < 0.0001) and LGR5 expression (from 0.30 ± 0.07‐fold of UD controls to 0.77 ± 0.06‐fold to of UD controls, *p* < 0.0001) in differentiated organoids (DO), suggesting an activation of the stem cell niche (Figure 3B,C). To validate the involvement of RET receptor for the GDNF signalling cascade, we used the specific RET receptor inhibitor BLU‐667. BLU‐667 significantly negated the effect of GDNF concerning Ki67 (0.12 ± 0.03‐fold, *p* < 0.0001) and LGR5 (0.36 ± 0.05‐fold, *p* < 0.0001) expression compared to controls effects (Figure 3B,C). In summary, these data indicate that GDNF administration results in enhanced wound healing in human organoids which was paralleled by increased Ki67 and LGR5 expression via RET receptor signalling.

### 2.4 GDNF effects on epithelial wound healing are RET‐ and Src kinase dependent in vitro

**FIGURE 4:**
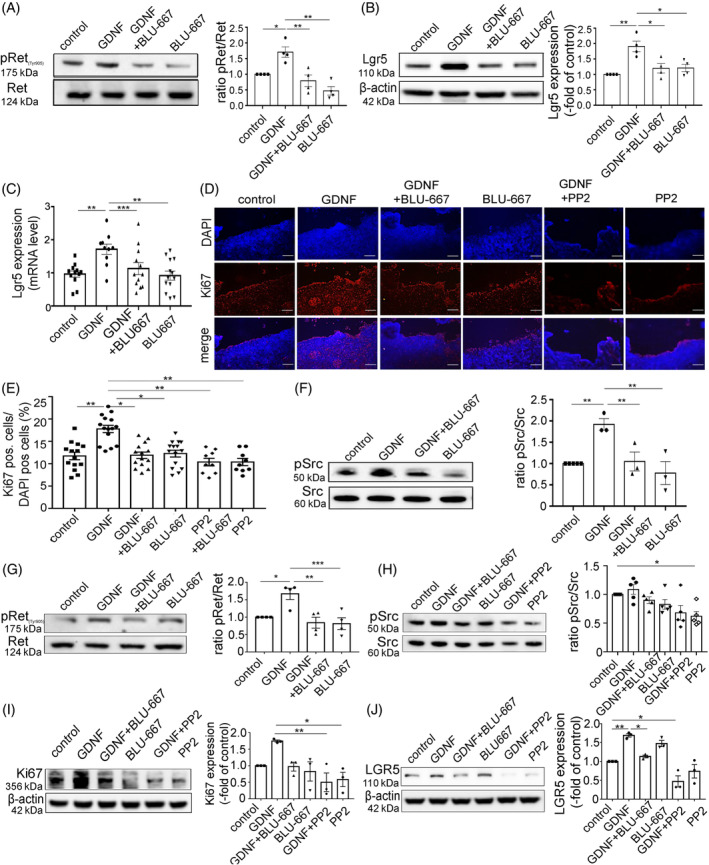
Increased cell proliferation mediated by GDNF via Ret and Src was abolished after application of RET inhibitor. (A, B) Representative Western blots in Caco2. The Cells were harvested 48 h after scratching and were in the respective conditions for this time (*n* = 4). Data are mean ± SEM (*n* = 4 experiments) and were analysed by 1‐way ANOVA followed by Tukey's post hoc testing; *p ≤ 0.05, **p ≤ 0.01.(C) qRT‐PCR measurements of Lgr5 expression in CaCo2 cells, the cells were harvested 48 h after scratching and were in the respective conditions for this; mean ± SEM (*n* >10) and were analysed by 1‐way ANOVA followed by Tukey's post hoc testing. *p ≤ 0.05. (D) Representative immunofluorescence staining of Caco2 scratches after 48 h; DAPI (blue) and Ki67 (red) and merge, Scale bar 50 μm. (E) Analysis of the proportion of Ki67‐positive cells in the total cell count in a defined image section. Data are mean ± SEM (*n* = 7) and were analysed by 1‐way ANOVA followed by Tukey's post hoc testing. **p ≤ 0.01. (F) Representative Western blots of Caco2 48 h after scratch. Data are mean ± SEM (*n* = 3) and were analysed by 1‐way ANOVA followed by Tukey's post hoc testing; *p ≤ 0.05, **p ≤ 0.01. (G–J) Western blots from murine organoids (*n* = 4 in G, H, I; *n* = 5 in J) after 24 h incubation with different mediators. Data are mean ± SEM (*n* = 4 experiments) and were analysed by 1‐way ANOVA followed by Tukey's post hoc testing. *p < 0.05, **p ≤ 0.01. All Western blot quantifications represent the OD of the protein of interest related to β‐actin or the phosphorylated protein as –fold of control. GDNF, glial cell line‐derived neurotrophic factor.

To further identify GDNF‐dependent signalling pathways in intestinal epithelium, we used differentiated Caco‐2 cells and murine organoids as described before.^9^ In the first step, increased phosphorylation of the RET receptor at Tyr905 was verified in Caco2 cells (Figure 4A). Combined treatment of GDNF with RET inhibitor (BLU‐667) blocked GDNF‐induced RET phosphorylation, whereas BLU‐667 had no obvious impact on RET phosphorylation under basal conditions. Since wound healing and cell proliferation occur both during would healing we assessed whether cell migration would be increased by GDNF together with the effects on cell proliferation by assessing whether focal adhesion kinase (FAK) was activated in response to GDNF. This resulted heterogenous effects with a trend towards increased pFAK‐levels in response to GDNF in Caco2 cells (Figure S2). Therefore, we concluded that GDNF effects observed in this study are primarily mediated by increased proliferation.

Comparable to the situation in vivo and in human organoids LGR5 protein levels were upregulated in Caco2 cells after GDNF application, which was blocked by co‐incubation with BLU‐667 in Western Blot analyses and in qRT‐PCR measurements (Figure 4B,C). Since a link between RET‐dependent signalling and downstream activation of SRC kinase has been reported previously in the context of increased epithelial proliferation,^13^ we used the SRC inhibitor PP2 to further test the involvement of SRC kinase on GDNF effects. After mechanical wounding, staining of Caco2 monolayers for Ki67 (Figure 4D) showed that the number of proliferating cells was significantly increased in GDNF‐treated monolayers compared to controls (10.57 ± 0.82% under control conditions, 13.95 ± 0.85% following GDNF treatment, *p* < 0.01). The GDNF‐induced increase of Ki67‐positive cells was attenuated when monolayers were co‐incubated with RET inhibitor BLU‐667 or SRC inhibitor PP2, whereas RET inhibition alone or PP2 alone did not change the number of Ki67‐positive cells compared to untreated controls (Figure 4E). To test whether increased cell proliferation would only occur at the edge of the wound healing sites or also in the follower cells we assessed the amount of Ki67 cells at different sites of the Caco2 monolayers. GDNF treatment led to a significant increase in the number of Ki67‐positive cells in the cells directly at the wound site 80.18 ± 4.72% (wound area) compared to the control 62.42 ± 2.72% (*p* < 0.05; Figure S1A,B). The effect was attenuated by inhibition with the RET inhibitor BLU‐667 (59.44 ± 4.45%; *p* < 0.01) and the PP2 inhibitor (47.38 ± 6.22%; *p* < 0.0001; Figure S1B). A comparable effect was observed in the following cells, although the overall number of proliferating cells was overall lower than in the cells adjacent to the wound area: Under control conditions 11.6 ± 0.54% were Ki67‐positive. Application of GDNF increased the number of Ki67‐positive cells to 26.91 ± 0.54% (*p* < 0.05; Figure S1B). BLU‐667(11.87 ± 1.89; *p* < 0.05;) and PP2 (11.07 ± 0.81; *p* < 0.05) inhibited GNDF‐induced effects of Ki67‐positive cells.

In line with this, increased phosphorylation of SRC kinase following GDNF application in Caco2 cells was present. This was inhibited by RET inhibitor BLU‐667 suggesting that SRC activation occurs downstream of GDNF‐mediated RET activation (Figure 4F). These observations were confirmed in murine organoids where increased RET and Src kinase phosphorylation by GDNF were attenuated by BLU‐667 (Figure 4G,H). Moreover, the impact of SRC inhibition on Ki67 expression was also examined in murine organoids. Western blots from murine organoids showed an increased Ki67 protein expression after GDNF application which was attenuated following simultaneous application of BLU‐667 or PP2. BLU‐667 or PP2 alone did not alter Ki67 protein expression compared to controls (Figure 4I). In line with the observations made in vivo, in human organoids and in Caco‐2 cells, increased LGR5 expression after GDNF treatment was attenuated using BLU‐667 and PP2 in murine organoids whilst the administration of the inhibitors alone did not show effects on LGR5 expression (Figure 4J). In summary, these observations show that GDNF effects on LGR5 and Ki67 expression are mediated via RET‐dependent activation of SRC kinase.

### 2.5 GDNF‐induced wound healing in vitro are RET‐ and src‐dependent

**FIGURE 5:**
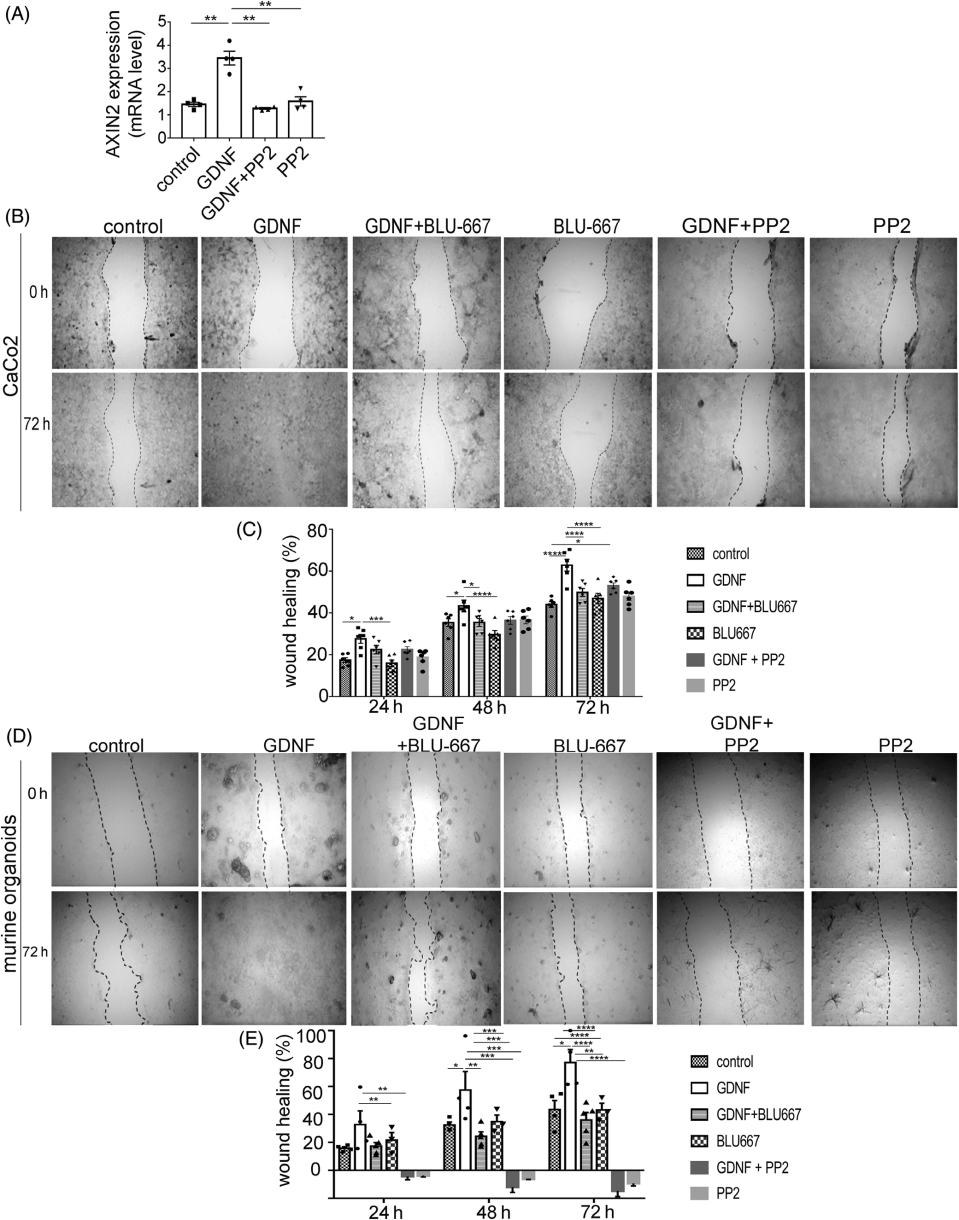
Epithelial wound healing by GDNF is RET‐ and src‐dependent. (A) qRT‐PCR measurements are shown for the expression of the AXIN2 in Caco2 cells under different conditions. Data are shown as mean ± SEM (*n* = 5 experiments) and were analysed by 1‐way ANOVA followed by Tukey's post hoc testing. *p ≤ 0.05. (B) Representative images of Caco2 wound healing assays under different conditions are shown for the wounding situation after wounding and after 72 h (*n* = 9). (C) Quantification of the wound healing assays Caco2 monolayers after 24, 48 and 72 h in relation to the initial wound area under the different conditions is shown. Data are shown as mean ± SEM (*n* = 9 experiments) and were analysed by 1‐way ANOVA followed by Tukey's post hoc testing. *p ≤ 0.05, **p ≤ 0.01, ****p ≤ 0.0001. (D) Representative images of two‐dimensionalized murine organoid wound healing assays are shown after wounding and after 72 h (*n* = 9). (E) The graph shows the quantification of the wound healing experiments in murine organoids. The relation of the the wound closure of the injured area after 24, 48 and 72 under the different conditions is shown as percentage of wound closure. Data are shown as mean ± SEM (*n* = 5 experiments) and were analysed by 1‐way ANOVA followed by Tukey's post hoc testing; *p ≤ 0.05, **p ≤ 0.01, ****p ≤ 0.0001.

Since intestinal stem cell proliferation is closely linked to a highly active Wnt signalling pathway, we determined whether Wnt signalling was activated in response to GDNF application. To test this, we assessed *AXIN2* expression in Caco2 cells using qRT‐PCR. Compared to untreated controls GDNF resulted in increased AXIN2 expression to 3.45 ± 0.3‐fold of controls which was blocked with PP2 to 1.27 ± 0.19‐fold co‐incubation with GDNF (Figure 5A). Finally, we used in vitro wound healing assays to test the functional relevance of the signalling pathways determined here. In wound healing assays in Caco2 monolayers, GDNF application accelerated wound closure compared to controls (Figure 5B). After 24 h, wound area closure amounted to 17.4 ± 2.71% under control conditions, which was significantly increased to 27.58 ± 2.71% of baseline measurements following GDNF application. This effect was reduced to 22.47 ± 2.63% of the baseline when the RET inhibitor Blu667 was applied together with GDNF. Consistent changes were observed at 48 and 72 h after wounding. The application of BLU‐667 alone did not have obvious effects on monolayer wound healing. Interestingly, the combined application of GDNF and Src inhibitor PP2 blocked GDNF‐induced accelerated wound healing, whereas PP2 alone had no effect on wound closure compared to controls (Figure 5B,C). Similarly, mouse organoid‐derived monolayers were seeded on plates, and wound‐healing assays were performed (Figure 5D). The quantification (Figure 5E) of wound closure showed a significantly faster closure of the monolayer after GDNF application compared to untreated controls. This was blunted when cells were co‐incubated with RET inhibitor BLU‐667 or SRC inhibitor PP2 comparable to the observations made in Caco‐2 cells. In summary, these experiments confirmed the functional relevance of RET‐dependent SRC kinase activation by GDNF to promote intestinal epithelial wound healing.

## 3 DISCUSSION

The present study demonstrates a new mechanism by which GDNF contributes to intestinal epithelial wound healing. GDNF restored mucosal healing in an inflammation recovery model and in biopsy‐based wounding assays in vivo. In vitro studies revealed that GDNF strongly activated RET/SRC‐dependent signalling which led to upregulation of LGR5 and to increased cell proliferation. This points to a novel role of RET receptor signalling in the context of intestinal stem cell activation and mucosal regeneration.

### 3.1 Induction of mucosal healing by GDNF is mediated by increased proliferation in vivo

Our data show a beneficial effect of GDNF on mucosal healing in two different in vivo models. As revealed by colonoscopy‐induced wounding assays in mice, GDNF resulted in faster wound closure compared to controls. This assay has been used before and has proven to be the current gold standard for assessing mucosal healing.^16^, ^17^ A comparable observation was made in the acute DSS colitis recovery model when the extent of mucosal lesions and overall inflammation was attenuated after the application of GDNF. This was also reflected by a faster recovery of the GDNF‐treated animals as represented by DAI. The observation that GDNF induces mucosal healing is novel since previous studies predominately focused on the restoration of intestinal barrier function in the acute DSS colitis model^9^, ^18^, ^19^ or reported a beneficial role of GDNF in the context of ischemia–reperfusion injury.^18^ In the present model, GDNF was applied therapeutically since DSS‐induced colitis had been induced first for 5 days before GDNF application which supports the notion that GDNF had regenerative effects rather than inhibitory effects on inflammation‐induced changes. It is interesting to note, that the effects of GDNF on cell proliferation were more pronounced in the distal colon than in the proximal colon.^20^, ^21^ This may be explained by a different extent of inflammation that is known to be stronger in the distal colon in DSS‐induced colitis. The observations made in vivo are supported by the in vitro effects where GDNF accelerated wound healing in an epithelial cell line as well as in human and murine intestinal organoids. On a mechanistic level, an increased number of proliferating cells, as revealed by enhanced Ki67‐staining in crypts and wound margins of the in vitro assays, was detected. The positive effect of GDNF is evidenced by the presence of Ki67‐positive cells in both the wound edge and follower cells, indicating its role in modulating cell proliferation. In our study, we observed Ki67‐positive cells in both leader and follower cells in Caco2 cells during wound repair. This contrasts with primary epithelial cells, where leader cells typically do not proliferate.^3^, ^22^ This discrepancy could be attributed to the cancerous nature of Caco2 cells, which exhibit dysregulated growth and overall increased proliferation. As cancer cells, including Caco2, may proliferate throughout the wound area, their altered regulatory mechanisms must be considered when interpreting these proliferation patterns in wound repair studies. Nonetheless, it has to be emphasised that mucosal healing always involves cell proliferation and migration at the same time.^23^ To assess whether cell migration would also be affected by GNDF we assessed phosphorylation patterns of focal adhesion kinase (FAK) as a read out for cell migration. According to Western blot analyses, we received heterogenous results showing at least a partial increase of pFAK in response to GDNF (Figure S2). Therefore, we concluded that the induction of cell proliferation rather than cell migration plays a dominant role when assessing the GDNF effects.

### 3.2 GDNF effects in intestinal enterocyte are RET‐dependent

GDNF is known to act via a multicomponent receptor system consisting of four GFRα (α1‐α4) subunits of the GDNF family receptors (GFR) and RET kinase.^24^ All of these receptors are not only present in the neuronal and endocrine system but are expressed in enterocytes.^12^, ^13^ Accordingly, inhibition of the RET tyrosine kinase using RET‐specific inhibitors such as BLU‐667 blocked previously observed effects of GDNF in cell culture experiments.^9^, ^12^, ^13^ Our present data confirm these findings since the application of RET inhibitor BLU‐667 in an enterocyte cell line and in organoids blunted all of the effects observed after GDNF application, including RET‐phosphorylation. This is important since it suggests that GDNF‐dependent increased cell proliferation is mediated through the RET pathway. In preliminary dose–response experiments, we had chosen the lowest dose possible of RET inhibitor BLU‐667 to minimise cross‐reactions on other tyrosine kinases, although this cannot be excluded completely. Attempts to knock out the RET receptor in intestinal organoids have not been successful in our hands so far since most of the cultures did not grow sufficiently.

Besides this, this raises the question of how increased proliferation by RET‐dependent signalling is activated in detail. In general, RET signalling cascade is involved in a variety of developmental processes in cells. Especially, mutations of RET are well known to be causative of malignant tumour development in the endocrine system and in lung cancer.^25^, ^26^, ^27^ In contrast, the potential physiological role of RET‐dependent signalling in intestinal epithelial cells has rarely been addressed in the past. Whilst a previous manuscript indicates that intestinal epithelial RET is required to maintain gastrointestinal motility in male animals by limiting peptide YY release,^28^ the observation of GDNF‐induced proliferation points to a novel role of epithelial RET receptor activation. This is supported by a previous observation made in drosophila where intestinal epithelial knock out of RET receptor demonstrated a reduced proliferation in intestinal precursor cells.^13^ Our current data confirm that RET‐induced proliferation is important in murine and in human intestinal epithelial cells.

### 3.3 RET activated SRC kinase induces LGR5 upregulation, which is required to promote proliferation

It is well known that the tyrosine kinase SRC is activated following phosphorylation and activation of the RET receptor.^13^, ^29^, ^30^ This is of great relevance since SRC kinase activation has been linked to increased proliferation by activation of Wnt/β‐catenin signalling.^13^, ^31^ RET‐dependent activation of SRC kinase in intestinal epithelial cells is consistent with our findings that GDNF increases phosphorylation of RET and SRC. Vice versa, RET inhibition using BLU‐667 blocked GDNF‐induced phosphorylation of SRC in Caco2 cells and in organoids. The involvement of SRC kinase as a downstream mechanism of GDNF‐induced activation of RET is shown by experiments with SRC kinase inhibitor which blunted both proliferation and upregulation of Lgr5 in the present study. This is supported by observations in which IEC‐specific ablation of SRC prevented the regeneration of intestinal crypts after the induction of DNA damage by irradiation.^32^ The SRC induced intestinal regeneration has been shown to be mediated by activation of RAS/MAPK and STAT3 signalling.^33^ Lgr5 upregulation that was found in all models tested here has been reported before, neither in the context of RET activation nor following activation of SRC kinase. This led to the question of whether this is just a phenomenon that occurs independently of proliferation to suggest the presence of an increased number of intestinal stem cells or whether this is critical to mediate activation of Wnt‐dependent signalling by RET in intestinal epithelial cells. In this context, Lgr5 is meanwhile widely accepted as an intestinal stem cell marker and its activation by R‐spondin1 is important to stimulate Wnt/β‐catenin signalling.^6^ In our experiments using human organoids GDNF was effective to activate proliferation even after Wnt‐depletion was carried out to induce organoid differentiation. This may be explained by the observation that epithelial cells in particular Paneth cells produce small amounts of Wnt in culture which may be sufficient to promote Wnt signalling by GDNF under these conditions.^34^ Another study demonstrated the positive effect of GDNF on cutaneous wound healing and increased hair follicle growth as well as hair follicle stem cell activation.^24^ The involvement of the Wnt/β‐catenin pathway can be assumed by the experiments in Caco2 cells where *AXIN2* was augmented following GDNF application, which may explain the strong effect on intestinal epithelial proliferation. The fact that both, RET and SRC kinase activation were effective to block GDNF‐induced increase of Axin2 supports the relevance of the pathway reported here.

The source of GDNF in the gut has been addressed by several previous studies where it has been reported that that GDNF in the gut is not only derived from enteric glial cells but also to a significant amount by smooth muscle cells^12^, ^35^ Furthermore, cell culture experiments revealed that enterocytes may also synthesise and secrete GDNF.^11^

### 3.4 Summary and outlook

Here, we demonstrate a novel mechanism of RET‐dependent activation of the intestinal stem cell niche, resulting in significantly enhanced cell proliferation and epithelial recovery. The upregulation of LGR5 by RET‐dependent mechanism may help to promote the regenerative response of the intestinal epithelium. From a translational point of view, the observed effects of GDNF add to previous observations in which loss of GDNF was associated with severe inflammation and reduced intestinal barrier function in patients with IBD.^9^ This can help to identify novel therapeutic strategies in the future.

## 4 MATERIALS AND METHODS

### 4.1 Antibodies and test reagents

Antibodies and test reagent used in this study are shown in Tables 1 and 2.

**TABLE 1:** Primary and secondary antibodies with dilutions used for western blotting (WB) and immunofluorescence staining (IS).

Antibody	Source	Catalogue number	WB	IS
Alexa Fluor 488	Thermo Fisher Scientific, Waltham, USA	A‐21201	n/a	1:200
Alexa Fluor 488	Thermo Fisher Scientific Waltham, USA	A‐21206	n/a	1:200
Cy3	Dianova, Hamburg, Germany	115–165‐003	n/a	1:600
Cy3	Dianova, Hamburg, Germany	111–165‐003	n/a	1:600
Ki67	Abcam, Cambridge, UK	ab15580	1:500	1:200
DAPI	Calbiochem, Darmstadt, Germany	268,298	n/a	1:1000
LGR‐5	BD‐Transductions, Laboratories, USA	30,007‐1‐AP	1:500	n/a
RET	Santa Cruz, Dallas, USA	sc‐101,423	1:200	n/a
Phospho RET (Tyr905)	Cell signalling, Danvers, USA	3221	1.500	n/a
Src (32G6)	Cell signalling, Danvers, USA	2123	1:1000	n/a
Phospho Src (Tyr416)	Cell signalling, Danvers, USA	2101	1:1000	n/a
E‐cadherin	Santa Cruz, Dallas, USA	sc‐101,580	1:200	1:400
pFAK	Cell signalling, Danvers, USA	3284S	1:1000	n/a
FAK	Cell signalling, Danvers, USA	3285S	1:1000	n/a

**TABLE 2:** Test reagent used in this study are shown including the concentration applied in vivo and in vitro, respectively.

Reagent	Source	Catalogue number	Applied concentration in vivo	Applied concentration in vitro
GDNF	PeproTech (Rocky Hill, USA)	450–44	100 ng/mL	100 ng/mL
RET‐Kinase Inhibitor (BLU‐667)	MedChemExpress (New Jersey, USA)	HY‐112301	n/a	70 ng/mL
Src kinase Inhibitor (PP2)	Abcam, (Cambridge, UK)	172,889–279	n/a	50 ng/mL

### 4.2 Animal experiments

Male C57BL/6J mice (aged 10–14 weeks, weight: 20–25 g, Janvier, 53,940 Le Genest Saint Isle, France) were used in this study. *Our study examined male mice because male animals exhibited less variability in phenotype*. Animals were maintained under specific‐pathogen‐free (SPF) conditions with regulated daylight (12:12 light/dark cycle), humidity (55 ± 10%) and temperature (22 ± 2°C). The mice were housed in pairs in sterile standard Makrolon type III cages on autoclaved wood shavings with autoclaved paper towel as nesting material and sterile environmental enrichment items: spinning wheel, shelter and nest box (PLEXX, Netherlands). They had access to autoclaved pelleted standard diet (#1320 M fortified, Altromin GmbH, Lage, Germany) and filtered municipal tap water in drinking bottles ad libitum. Cages were kept inside ventilated cabinet Scantainer (Scanbuhr AS, Lellingegaard, Denmark).

### 4.3 Murine dextran sodium sulphate (DSS) colitis recovery model

As described previously, the DSS colitis recovery model, a well‐established in vivo model for intestinal inflammation was used.^36^ This involved the administration of 2.5% DSS (DSS, 40–50 kDA, Biozol, Eching, Germany) dissolved in drinking water for 5 days, followed by 5 days of DSS‐free drinking water. Weight loss was assessed daily as well as stool consistency, and presence of blood in the stool (Haemocult, CARE diagnostic, Vörde, Germany) were assessed daily. These parameters were semi‐quantitatively scored with scores of 0–4 as published previously.^37^ The individual scores were averaged and constituted the disease activity index (DAI) in which increasing values reflect the severity of colitis. After 5 days of DSS application, animals were randomly assigned to the control group (*n* = 13) and the GDNF group (*n* = 13) in which recombinant GDNF was applied intraperitoneally (i.p.) at 5 μg/kg body weight in 100 μL NaCl 0.9% for 4 days during the recovery phase. The concentration of GDNF applied has been established previously.^9^ Control animals received 100 μL NaCl 0.9% i.p. for 4 days.

After the end of experiment, mice were euthanized, colon length was measured. Colon was harvested and cut longitudinally into two pieces. One part was fixed in 4% paraformaldehyde, embedded in paraffin and sectioned. Sections of the entire colon, mounted in swiss rolls and cut to a thickness of 2 μm, were stained with haematoxylin and eosin (H&E) to evaluate colonic mucosal injury. The slides were scanned with an Pannoramic Scan II (3DHistech, Budapest, Hungary). Two investigators (MH and MK) used CaseViewer (3DHistech, Budapest, Hungary) to score the extent of inflammation in colon tissue sections stained with H&E. The scoring system ranged from 1 (no inflammation) to 8 (severe inflammation). The scoring system used was based on the extent of inflammatory cell infiltration (none = 1, mucosal infiltration = 2, submucosal infiltration = 3, transmural infiltration = 4) and the severity of epithelial damage (no epithelial damage = 1, focal lesions = 2, multiple lesions = 3, extended ulcerations = 4). This resulted in a total scoring range of 2–8 per mouse. The other half of the colon was lysed and homogenised with a Tissue Lyzer (Quiagen, Hilden, Germany) in SDS lysis buffer and used for Western Blot analysis or stored in RNA later (Sigma, Taufkirchen, Germany) for qRT‐PCR analyses as described previously.^9^

### 4.4 Murine mucosal wound healing assay

To assess intestinal epithelial wound healing in mechanically generated wounds, the biopsy‐based wound healing assay was used in C57BL/6J mice as already described in detail before.^36^ For the in vivo biopsy‐based mucosal wound healing model, a high‐resolution video endoscope (Coloview Veterinary Endoscope, Karl Storz, Tuttlingen, Germany) equipped with a biopsy forceps was used to induce standardised injuries to the colonic mucosa at three to five independent sites along the dorsal wall of the distal colon in isofluran (cpPharma, Burgdorf, Germany) anaesthetised mice. Wound healing was assessed at 24‐ and 72‐h post‐injury by re‐endoscopy using high‐resolution images (1024 × 768 pixels) on a flat‐panel colour monitor. Each wound region was photographed, and wound areas were measured using ImageJ (Rasband, W.S., ImageJ, Bethesda, Maryland, USA.) In each experiment, three to five wounds per animal were quantified by two independent investigators (MK and MH).

After mucosal injury had been carried out, animals were randomly assigned to the control group (*n* = 9) and the GDNF group (*n* = 8) in which recombinant GDNF was applied at 5 μg/kg body weight in 100 μL NaCl 0.9% i.p. daily. Control animals received 100 μL NaCl i.p. for the duration of the experiments. After 72 h post‐injury the experiments were terminated and colon tissue was harvested by punch biopsies from wounded areas and from non‐wounded epithelium in the same animals, fixated in 4% paraformaldehyde, embedded in paraffin and sectioned or fixed in RNA later for quantitative real‐time (qRT) PCR, respectively.

### 4.5 Caco2 cell culture

As described previously,^11^ Caco2 cells were cultured in DMEM supplemented with Penicillin–Streptomycin and 10% FCS. For passaging, cells at 80% confluency were treated with 1% EDTA in Dulbecco's Phosphate Buffered Saline (DPBS) and dissolved using Trypsin–EDTA. After centrifugation, the pellet was resuspended in DMEM medium.

### 4.6 Human intestinal organoid cultures

Organoid generation and culture was described in detail previously.^15^ In brief, human full‐wall tissue samples were obtained from patients with an indication for surgical bowel resection (e.g., for colon carcinoma) from a healthy resection margin (small intestine or colon). Prior to surgery, all patients gave written informed consent for sample collection. This was obtained via the broad consent of the medical faculty of the University of Wuerzburg. The Ethical Board of the University of Wuerzburg gave ethical approval (proposal numbers 46/11, 113/13, 42/16, amendment 2020, 171/22). After harvesting, tissue samples were immediately placed in Hank's Balanced Salt Solution (HBSS; Sigma‐Aldrich) supplemented with 1% Antibiotic‐Antimycotic 100× (Gibco, Carlsbad, California), HBSS/a‐a for transportation on ice. After washing the sample three times with PBS, the mucosa was dissected, mucus and villi were scraped off gently with glass slides and the mucosa was cut into strips. The mucosa was washed in HBSS/a‐a by vortexing 5 s and then digested in HBSS/a‐a supplemented with 2 mM EDTA under constant rotation for 30 min at 4°C. After digestion, the mucosa was washed by inverting slowly in fresh HBSS/a‐a. The mucosa was then transferred into 10 mL HBSS/a‐a supplemented with 1% Gentamicin (Genaxxon bioscience, Ulm, Germany) and gently shaken for 10 s to extract crypts. After shaking and repeated washing, the pellet was resuspended in 4°C cold mixture of 50% Growth Factor Reduced Basement Membrane Matrix (Corning, Corning, NY, United States) plus 50% IntestiCult Organoid Growth Medium Human (Stemcell Technologies, Vancouver, Canada) and seeded as 5–10 μL droplets in multiwell plates. After the gel mixture solidified upside down for 20–30 min at 37°C, the plate was reversed and 37°C warm IntestiCult supplemented with 10 μM Y27632 dihydrochloride (Tocris Bioscience, Bio‐Techne, Bristol, United Kingdom) was added. Medium was changed every 2–3 days using IntestiCult.^9^, ^12^, ^38^ Organoid cultures were passaged and seeded as described before in detail.^15^

### 4.7 Murine organoid generation

Murine organoid generation was carried out similarly to human organoids. The tissue was transferred into 20 mL of 4°C cold PBS, vortexed for 5 s and the supernatant discarded. This washing step was repeated until the supernatant was clear of cell debris. The tissue was then digested in PBS containing 2 mM ethylenediaminetetraacetic acid (EDTA) (Sigma‐Aldrich, St. Louis, MO, USA) for 30 min with gentle rotation. Following this, the tissue was washed in PBS by gently inverting the tube five times. The mucosa was transferred into a new tube with 10 mL PBS and shaken for 10 s manually, repeating this procedure two times with fresh tubes. The crypts were checked under a microscope, and the supernatants with the most viable crypts were pooled and centrifuged at 350 g for 3 min. The pellet was washed in 10 mL basal medium, Dulbecco's Modified Eagle's Medium (DMEM)‐F12 Advanced (Invitrogen, Carlsbad, CA, USA) supplemented with 1× N2, 2× B27, 1× Anti‐Anti, 10 mM HEPES, 2 mM GlutaMAX‐I (all from Invitrogen, Carlsbad, CA, USA) and 1 mM N‐acetylcysteine (Sigma‐Aldrich, St. Louis, MO, USA). After centrifugation at 300 g for 3 min, the cell pellet was resuspended in cold Growth Factor Reduced Basement Membrane Matrix (Corning, Corning, NY, USA) Drops of 5–10 μL were seeded in a multiwell plate and incubated at 37°C upside down for 20–30 min until the gel mixture solidified. The culture medium consisted of 50% fresh basal medium and 50% Wnt3A‐conditioned medium, with the addition of the following growth factors: 500 ng/mL hR‐Spondin 1, 50 ng/mL human Epidermal Growth Factor (hEGF) (both PeproTech, Rocky Hill, NY, USA), 100 ng/mL recombinant Noggin (PeproTech, Rocky Hill, NY, USA), 10 mM Nicotinamide, 10 μM SB202190, 10 nM [Leu15]‐Gastrin I (all three from Sigma‐Aldrich, St. Louis, MO, USA), 500 nM A83‐01 (Tocris Bioscience, Bristol, UK) and 500 nM LY2157299 (Axon MedChem, Reston, VA, USA). Immediately after splitting, the medium was supplemented with 10 μM Y27632 dihydrochloride (Tocris Bioscience, Bio‐Techne, Bristol, UK) and the cells were maintained as organoid cultures for 8–10 weeks.

### 4.8 ECIS‐based wound healing assay

The ECIS 1600R (Applied BioPhysics, Troy, NY, USA) was used to assess epithelial wound healing of 250 μm wounds induced in two‐dimensionalized human organoid monolayers on wound electrode arrays.^15^ Measurements were started after reaching confluency. The monolayers of intestinal organoids were wounded (10 s, 2000 μA, 40,000 Hz) and changes of transepithelial electrical resistance (TER) were assessed in the time course of epithelial recovery. TER values were normalised to baseline values before wounding.

### 4.9 Mechanical wound‐healing assay in organoids and Caco2 cells

2D organoid monolayers were cultured as described before.^15^ Organoids were washed once with Basalmedium (BM) and resuspended in 1 mL TrypLE Express Enzyme. Resuspended organoids were kept at 37°C for 5 min and split mechanically, using a double tip, to receive a single cell solution. The splitting process was stopped by adding 1 mL of BM. Cells were washed again and resuspended in culture medium for murine intestinal organoids.

For seeding, 12‐well TPP® tissue culture plates (TPP, Trasadingen, Switzerland) were used, previously coated with 10% Geltrex LDEV‐Free Reduced Growth Factor Basement Membrane Matrix (Gibco) in BM. The solution was kept for 30 min inside the wells. After removal, the surface was dried at room temperature and the single cell solution was added. After reaching confluency, murine intestinal organoids experiments were conducted as described before.^15^ Caco2 cell monolayers or 2D organoid monolayers were cultured in 6 well or 8 well plates, respectively (Ibidi, Gräfelfing, München). Confluent cell monolayers were scratched using a 10 μm pipette described previously.^11^, ^36^ All scratches were performed by the same blinded investigator and the medium was changed. Images of the wounds were taken at 0‐, 24‐, 48‐ and 72‐h post‐scratch, and wound closure was quantified using ImageJ software, compared to the measured wound area of the initial (*t* = 0) scratch area.^11^ Monolayers were treated with GDNF, BLU‐667, PP2 or a combination as indicated below.

### 4.10 Quantitative real‐time PCR (qRT‐PCR)

RNA samples were prepared from human or murine tissue samples, Caco2 cells as well as murine organoids using Trizol and cDNA was synthesised with iScriptTM cDNA Synthesis Kit (Biorad, Munich, Germany). Quantitative PCR was performed using MESA GREEN qPCR MasterMix Plus for SYBR® Assay No ROX (Eurogentec, Cologne, Germany) on the CFX96 Touch Real‐Time PCR Detection System (Biorad, Munich, Germany). Gene expression was analysed via the BioRad CFX Manager software with β‐actin as a reference gene. All measurements were performed in duplicates at 60.0°C annealing temperature. Primers were applied at a concentration of 5 μM. Primer sequences are listed in Table 3.

**TABLE 3:** Primers used for quantitative real‐time PCR.

Primer	Species	Source	Primer sequences 5′‐3′
*MKI67* forward	Human	Eurofins	CCT GTG AGG CTG AGA CAT GG
*MKI67* reverse	Human	Eurofins	CCC TCA CTC TTG TCA GGG TC
*LGR5* forward	Human	Eurofins	CAG GCC GTC TGT GAT CAG TT
*LGR5* reverse	Human	Eurofins	GCA GCC TGA CAA ACT GGG TA
*AXIN2* forward	Human	Eurofins	CCA CAC CCT TCT CCA ATC C
*AXIN2* reverse	Human	Eurofins	TGC CAG TTT CTT TGG CTC TT
*ACTB* forward	Human	Eurofins	CCT CGC CTT TGC CGA TCC
*ACTB* reverse	Human	Eurofins	CGC GGC GAT ATC ATC ATC C
*Mki67* forward	Mouse	Eurofins	TGCAGCAGATGGAACTAGGC
*Mki67* reverse	Mouse	Eurofins	CACTGTGATCTTCCAGCGGT
*Actb* forward	Mouse	Eurofins	GGT GCT TGT CTC ACT GAC CGG C
*Actb* reverse	Mouse	Eurofins	TTC TCC GGT GGG TGG CGT GA

### 4.11 Western blot

For Western blotting, cell lines, mouse colonic tissue and organoids were lysed, homogenised, and protein quantitated using a BCA assay kit (Thermo Scientific, Massachusetts, USA) as previously described.^15^ Cells were lysed in a buffer, homogenised, and protein quantification was done using a BCA assay kit (Thermo Scientific, Massachusetts, USA). SDS gel electrophoresis was followed by protein transfer to a nitrocellulose membrane and overnight incubation with primary antibodies (Table 1). Secondary antibodies were applied for 1 h at room temperature, and signals were detected using SuperSignal West Pico PLUS Chemiluminescent Substrate (Thermo Scientific, Massachusetts, USA). Optical density measurement and normalisation to β‐actin were performed using ImageLab (Hercules, California, U.S.).

### 4.12 Immunostaining

The immunostaining was carried out as previously described.^38^ Mouse specimens were paraffin‐embedded and sectioned into 1 μm slices. Organoids were released from Matrigel, fixed and paraffin‐embedded. Monolayers and tissue slides were incubated overnight at 4°C with primary antibodies specified in Table 1. Secondary antibodies, including Cy3‐ or Alexa Fluor 488 labelled goat anti‐mouse or goat anti‐rabbit (Dianova, Hamburg, Germany), were utilised. Representative images were documented using a confocal microscope (Leica LSM 780; Zeiss, Oberkochen, Germany).

### 4.13 Statistics

Statistical analysis was performed using Prism (GraphPad Software, La Jolla, CA, USA). Data are presented as means ± Standard error (SEM) Statistical significance was assumed for *p* < 0.05. Paired Student's *t*‐test or Mann–Whitney‐U‐test was performed for two‐sample group analysis after checking for a Gaussian distribution through Shapiro–Wilk‐test. Analysis of variance (ANOVA) followed by Tukey's multiple comparisons or Šίdák's multiple comparisons test and Bonferroni correction was used for multiple sample groups. Kruskal‐Wallis‐Test was used for data not matching Gaussian distribution respectively. The tests applied for each of the different experiments are indicated in the figure legends.

### 4.14 Study approval

All animal experiments were reviewed and approved by the approval authority (licence number: 55.2–2532.2‐852) in accordance with the German Animal Welfare Act and the Directive 2010/64/EU of the European Parliament on the protection of animals used for scientific purposes. Care and use of male C57BL/6J mice (aged 10–14 weeks, weight: 20‐25 g, Janvier, 53,940 Le Genest Saint Isle, France) were in accordance with the German Animal Welfare Act and the Directive 2010/64/EU.

For human organoid generation samples were obtained from patients with an indication for surgical bowel resection (e.g., for colon carcinoma) from a healthy resection margin (small intestine or colon). Prior to surgery, all patients gave written informed consent for sample collection. This was obtained via the broad consent of the medical faculty of the University of Wuerzburg. The Ethical Board of the University of Wuerzburg gave ethical approval (proposal numbers 46/11, 113/13, 42/16, amendment 2020, 171/22).

## AUTHOR CONTRIBUTIONS

**MH**: Designing research studies; conducting experiments; acquiring data; analysing data and writing the manuscript. **NB**: designing research studies; conducting experiments; acquiring data; analysing data and writing the manuscript. **MK**: conducting animal experiments; acquiring data; analysing data and writing the manuscript. **AL**: conducting organoid experiments; acquiring data; analysing data. **TS**: conducting organoid experiments; acquiring data; analysing data. **CK**: conducting in vitro cell culture and organoid experiments; acquiring data; analysing data. **MM**: conducting in vitro cell culture and organoid experiments; acquiring data; analysing data. **CO**: designing animal studies; conducting animal experiments; acquiring data; analysing data. **CTG**: interpreting data; providing intellectual support. **KK**: designing organoid studies; analysing data. **SF**: designing research studies; conducting animal experiments; acquiring data; analysing data and writing the manuscript. **NS**: designing and supervision of all studies; analysing and interpreting data; writing the manuscript.

## FUNDING INFORMATION

This work was supported by the Deutsche Forschungsgemeinschaft [DFG SCHL1962/8–1 to NS, KE 2402/3–1 to MK] and the Interdisziplinäre Zentrum für Klinische Forschung (IZKF) [Z‐3R/3 to SF].

## CONFLICT OF INTEREST STATEMENT

The authors have declare no conflict of interest.

## ETHICS STATEMENT

All applicable international, national, and/or institutional guidelines for the care and use of animals were followed. Informed consent was obtained from all individual participants involved in the study.

## Supporting information


**Figure S1.** Quantification of Ki67 staining in cells at the edge of the healing wound and in follower cells.
(A) Schematic structure of the Ki67 scratch evaluation in Caco2 cells after 48 h treatment. The cells situated at the periphery of the wound area (blue) and those located behind the subsequent cells (yellow) were analysed separately.
(B) Quantification of the Ki67 wound healing assays in Caco2 monolayers after 48 h. There is an increased number of Ki67 positive cells in the wound cells as well as in the following cells after GDNF treatment for 48 h. This positive effect compared to the control was reversed with RET inhibitor and PP2. Data are shown as mean ± SEM (*n* = 6 experiments) and were analysed by 1‐way ANOVA followed by Tukey's post hoc testing; *p ≤ 0.05, **p ≤ 0.01, ****p ≤ 0.0001.
**Figure S2.** pFAK/FAK expression after GDNF treatment.
(A) Representative Western blots in Caco2. The cells were harvested 48 h after scratching and were in the respective conditions for this time (*n* = 5).
(B) The graph shows the Quantification of western blots. Data are mean ± SEM (*n* = 5 experiments) and were analysed by 1‐way ANOVA followed by Tukey's post hoc testing; *p* = 0.0129.

## Data Availability

There are no restrictions on data availability. The supporting data values are available in supplemental material. Additional data are available by request to the corresponding author.
