# Supplementary material for: Glial cell line derived neurotrophic factor (GDNF) induces mucosal healing via intestinal stem cell niche activation
Source: Cell Prolif. 2024 Nov 28;58(2):e13758. doi: 10.1111/cpr.13758 (PMC11839185; doi:10.1111/cpr.13758)
Supplement: Supplementary file 1 — Figure S1. Quantification of Ki67 staining in cells at the edge of the healing wound and in follower cells. (A) Schematic structure of the Ki67 scratch evaluation in Caco2 cells after 48 h treatment. The cells situated at the periphery of the wound area (blue) and those located behind the subsequent cells (yellow) were analysed separately. (B) Quantification of the Ki67 wound healing assays in Caco2 monolayers after 48 h. There is an increased number of Ki67 positive cells in the wound cells as well as in the following cells after GDNF treatment for 48 h. This positive effect compared to the control was reversed with RET inhibitor and PP2. Data are shown as mean ± SEM (n = 6 experiments) and were analysed by 1‐way ANOVA followed by Tukey's post hoc testing; *p ≤ 0.05, **p ≤ 0.01, ****p ≤ 0.0001. Figure S2. pFAK/FAK expression after GDNF treatment. (A) Representative Western blots in Caco2. The cells were harvested 48 h after scratching and were in the respective conditions for this time (n = 5). (B) The graph shows the Quantification of western blots. Data are mean ± SEM (n = 5 experiments) and were analysed by 1‐way ANOVA followed by Tukey's post hoc testing; p = 0.0129. [file CPR-58-e13758-s001.pptx]

## Slide 1
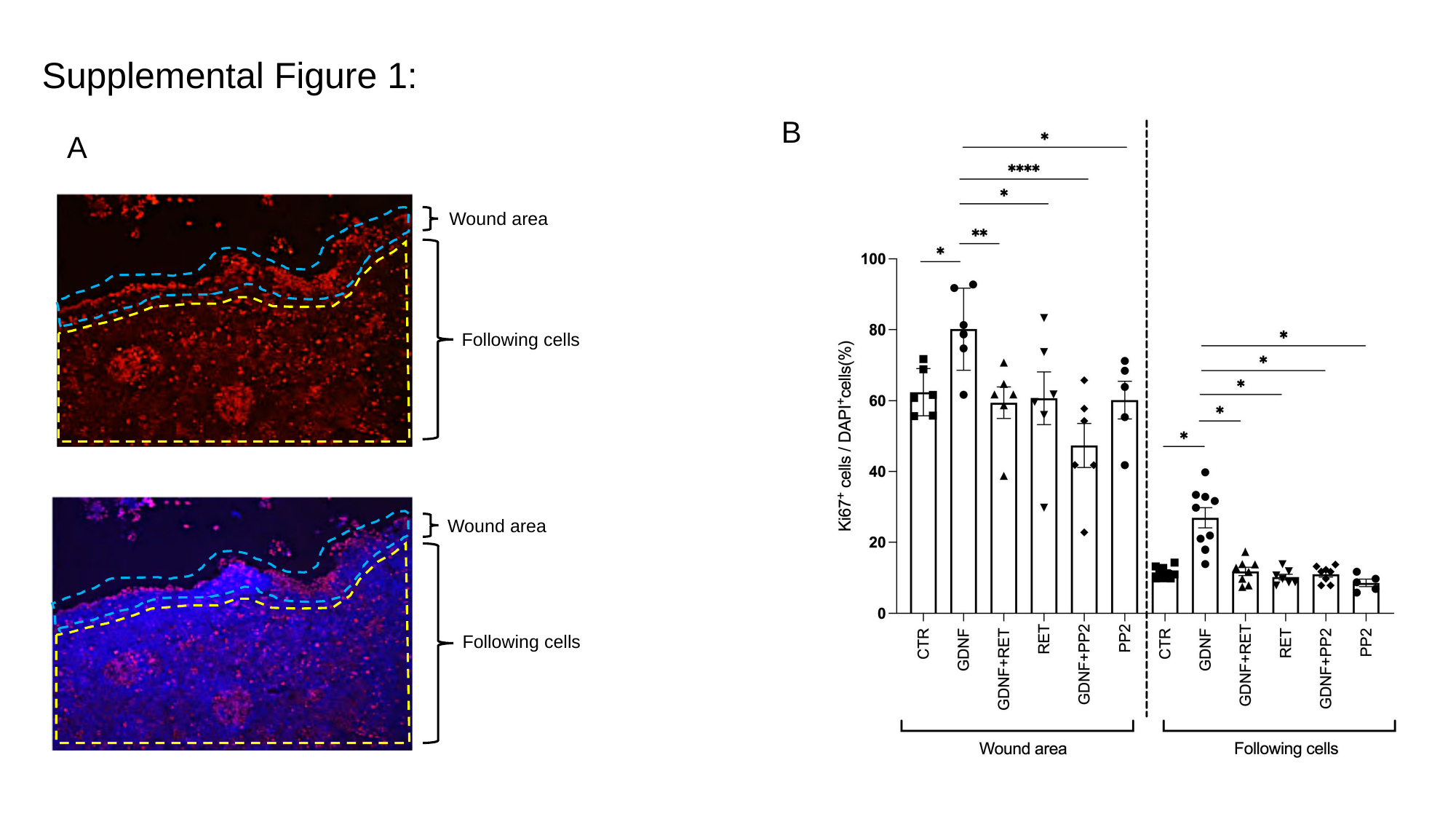

# Supplemental Figure 1:
B
A
Wound area
Following cells
Wound area
Following cells

## Slide 2
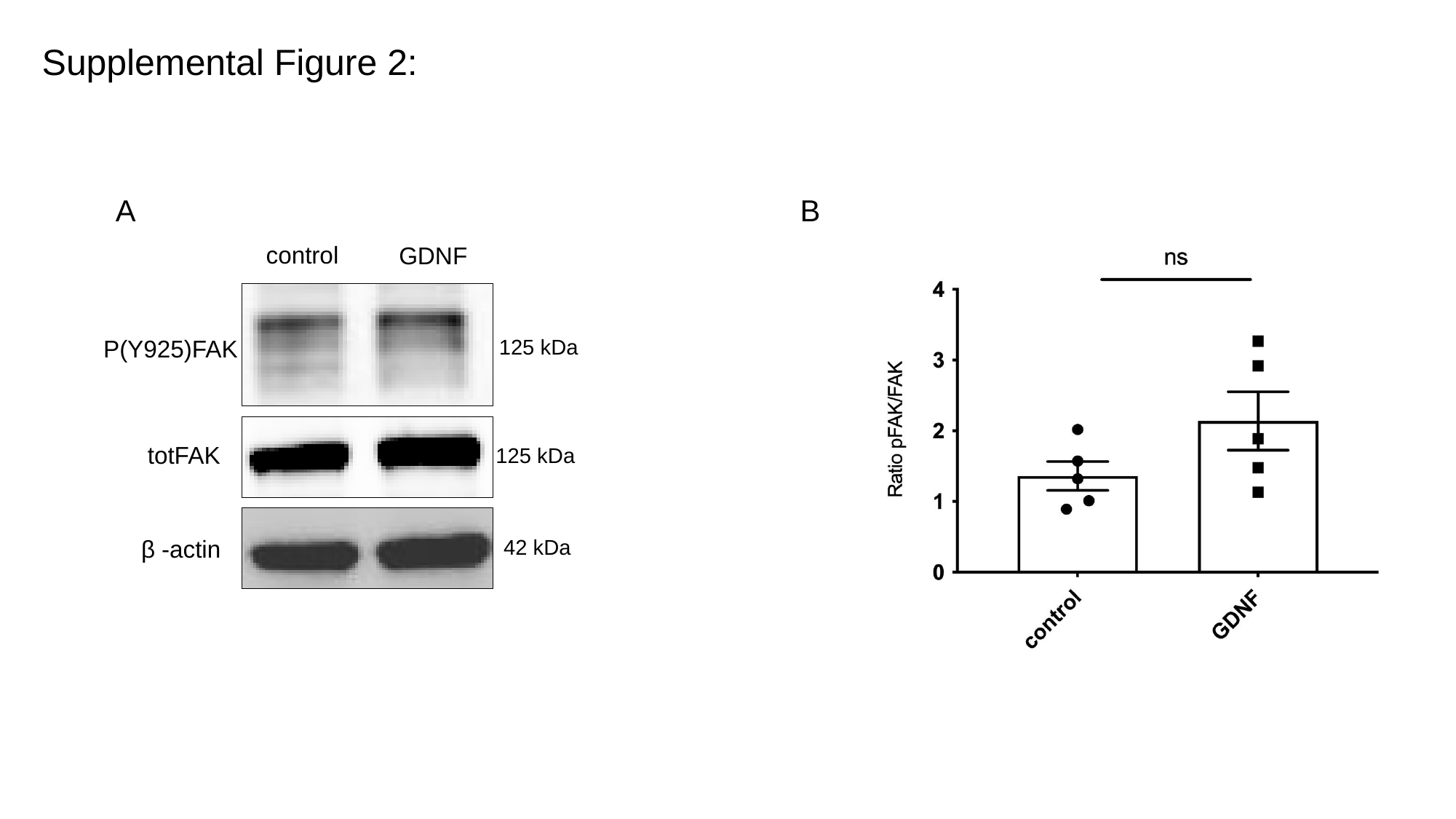

# Supplemental Figure 2:
A
B
control
GDNF
P(Y925)FAK
totFAK
β -actin
125 kDa
125 kDa
42 kDa
